# Tolerability of Surgery by Patients With Third Primary Lung Cancer After Second Primary Lung Cancer Surgery

**DOI:** 10.1111/1759-7714.70187

**Published:** 2025-11-16

**Authors:** Hideomi Ichinokawa, Kazuya Takamochi, Yukio Watanabe, Mariko Fukui, Aritoshi Hattori, Takeshi Matsunaga, Kenji Suzuki

**Affiliations:** ^1^ Department of General Thoracic Surgery Juntendo University Shizuoka Hospital Shizuoka Japan; ^2^ Department of General Thoracic Surgery Juntendo University Hospital Tokyo Japan

**Keywords:** computed tomography, lung cancer, prognosis, pulmonary fistula, surgery

## Abstract

**Purpose:**

This study aimed to clarify the incidences and treatments of enlarged nodules detected using imaging findings during follow‐up, surgical outcomes, and prognosis for patients who underwent a second surgery for primary lung cancer.

**Methods:**

We included 236 patients who underwent a second surgery for primary lung cancer (> 2 years between first and second surgeries). Group A comprised 205 patients without nodules. Group B comprised 31 patients with nodules that had grown and metachronous lung cancer was suspected; subgroup B1 underwent surgery (23 patients) and subgroup B2 did not (8 patients).

**Results:**

The incidences of new or enlarged intrapulmonary nodules following the second surgery were 0.0091, 0.015, 0.020, 0.024, and 0.029 after 1, 2, 3, 4, and 5 years, respectively. For the third surgery procedure, 83% of the patients underwent segmentectomy and wide‐wedge resection. Ten patients (44%) experienced complications associated with the third surgery including prolonged pulmonary fistulas in 6 patients (26%). The B1 subgroup 30‐day mortality rate after the third surgery was 4.3% (1/23). The 1‐, 2‐, 3‐, 4‐, and 5‐year survival rates after the second surgery in subgroup B1 were 95.7%, 86.3%, 80.9%, 80.9%, and 60.0%, respectively. No significant difference was observed in prognosis between the B1 and B2 subgroups (*p* = 0.11).

**Conclusion:**

Surgery can be considered effective for third primary lung cancer, notwithstanding associated complications, perioperative mortality, and prognosis.

## Graphical abstract

The incidence of new or enlarged intrapulmonary nodules after the second surgery was 0.0091, 0.015, 0.020, 0.024, and 0.029 after 1, 2, 3, 4, and 5 years, respectively.

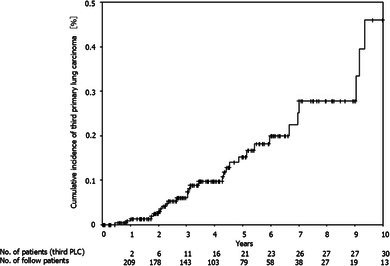

## 1 Introduction

Recently, although the number of lung cancer cases has increased, the number of long‐term survivors has also increased owing to improved treatment outcomes for lung cancer [1, 2]. However, the number of patients with metachronous lung cancer and new lung lesions detected during follow‐up after the initial lung cancer surgery has been rising, with the frequency of recurrence estimated to be 1%–2% [3]. Since 2010, patients with second metachronous lung cancer have had a good prognosis following surgery [4, 5, 6, 7, 8]. However, validated research has not been reported regarding surgery or treatment for suspected primary lung cancer with an enlarged intrapulmonary nodule on chest computed tomography (CT) during follow‐up after the second surgery. The first and second lung cancer surgery details, site and degree of progression for the first and second lung cancers, and patient respiratory function are often constraints in opting for a third surgery.

In this study, we investigated the incidences of enlarged nodules on imaging findings during follow‐up, treatment strategies, surgical outcomes, and prognoses for patients who underwent surgery for second primary lung cancer.

## 2 Methods

### 2.1 Study Population

**FIGURE 1:**
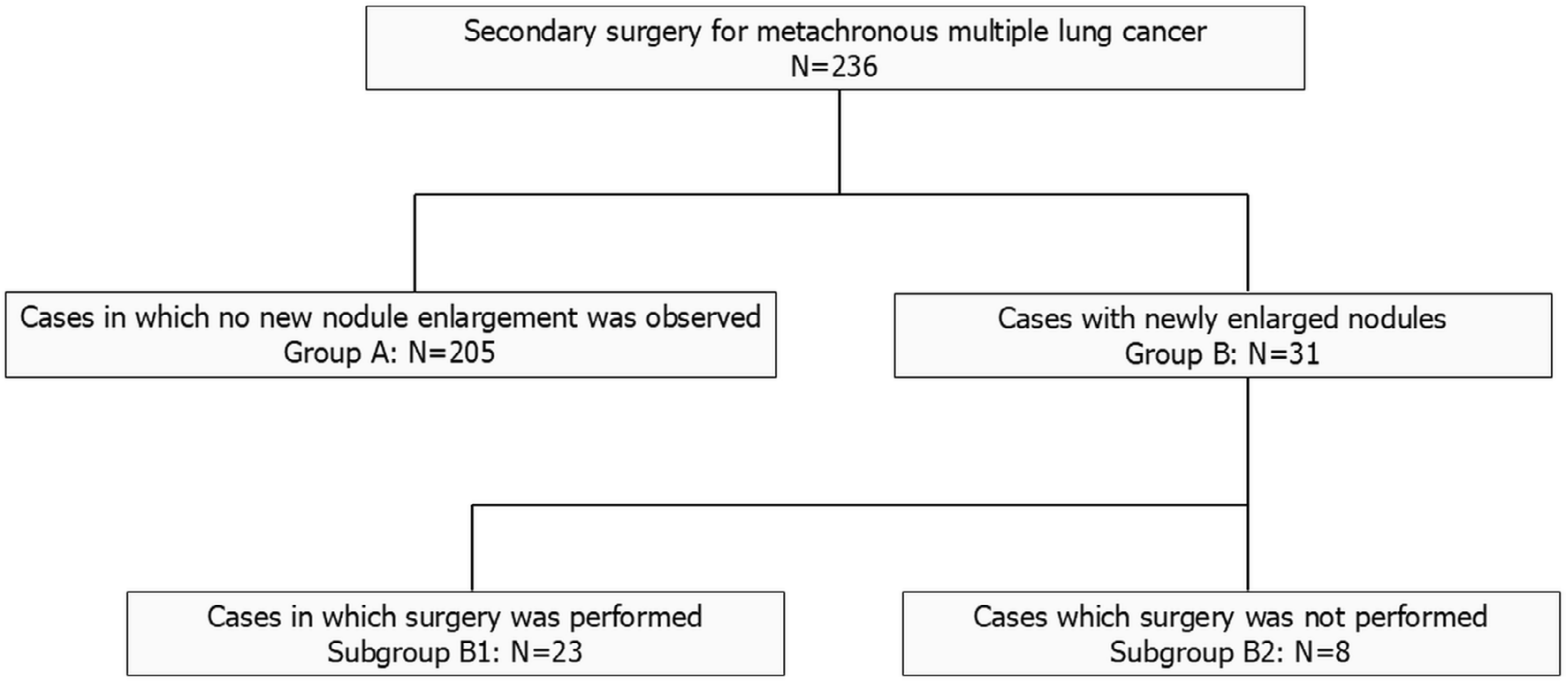
Patient distribution in the study.

This retrospective study was approved by the Ethics Committee of our institution (E24‐0349). Figure 1 illustrates the study details.

We retrospectively analyzed 5138 patients who underwent curative surgery for primary lung cancer at our hospital between April 2008 and December 2022 and identified 236 patients who underwent surgery for metachronous second primary lung cancer. The patients were followed up after surgery and categorized into group B, comprising those who were clinically considered to have a third metachronous primary lung cancer (31 patients), or group A, comprising those who did not have any new intrapulmonary nodules (205 patients). In group B, the patients were further categorized into subgroup B1, including those who underwent a third surgery for the nodules (23 patients) and subgroup B2 including those who did not (8 patients). The patients in subgroup B2 did not undergo surgery because the attending physician determined two patients as being ineligible for surgery and the remaining six patients did not choose surgery. Regarding the treatment plan for subgroup B2, six patients were in the observation group, two patients were in the stereotactic body radiotherapy (SBRT) group, and none of the patients chose radiofrequency ablation (RFA).

We analyzed the following clinical background characteristics and peri‐ and postoperative results: age at second and third surgery, sex, preoperative comorbidities, smoking history, pack‐year smoking, duration between the first and second surgery, respiratory function (vital capacity [VC]), %VC, forced expiratory volume in 1 s (FEV1.0), FEV1.0%, % predicted FEV1.0 (%FEV1.0), first and second surgical procedures, third intraoperative features (surgical site, surgical procedures, lymph node dissection, operation time, and intraoperative blood loss), histopathological findings, pathological stage, length of hospital stay, Clavien–Dindo grade ≥ 2 postoperative complications, presence or absence of recurrence, and treatment method recurrence. We also evaluated surgical morbidity and mortality rates within 30 and 90 days after surgery.

### 2.2 Operative Procedure and Follow‐Up

The attending physician decided the surgical procedure to be used based on the disease extent. In the first and second surgeries, the surgical procedure was preferably lobectomy; however, wide‐wedge resection, segmentectomy, lobectomy, pneumonectomy, or completion pneumonectomy (CP) was performed as necessary depending on the location of the primary tumor and metastatic lymph node infiltration.

The postoperative follow‐up period at our hospital is 10 years. The patients were followed up every 3 months for approximately 5 years after surgery and every 6 months after the first 5 years. The examination items were subjective symptoms, physical findings, blood tumor markers, and X‐rays. Chest CT was performed once a year. If lung cancer was suspected during follow‐up, head CT, magnetic resonance imaging, or positron emission tomography‐computed tomography (PET‐CT) and a whole‐body examination are performed. The median observation period following the second surgery was 1303 days.

### 2.3 Definition of Metachronous Lung Cancer

We performed a comprehensive diagnosis of metachronous lung cancer based on the diagnostic criteria proposed by Martini and Melamed [9] and the American College of Chest Physicians in 2013 presented by Kozower et al. [10]: (1) the interval between the first and second surgeries was > 2 years, (2) cancer histological types in the first and second surgeries were the same, (3) first and second lung cancers originated from different lobes of the lung, and (4) cancer was not detected in the common lymphatic pathway according to the pathological results of the first and second surgeries. In addition, patients without evidence of a histological diagnosis were clinically diagnosed with metachronous lung cancer based on the following guidelines: (1) a radiologist's diagnosis of primary lung cancer as suspicious based on imaging findings, (2) the nodule showed a slow growth trend for > 6 months after the clinical course, (3) no lesions were suspected to be malignant other than the lung lesion found following whole‐body CT or PET‐CT examination, and (4) a new nodule appeared after the previous surgery was visualized as an isolated shadow without lymph node swelling.

## 3 Statistical Analyses

Descriptive statistics were used to assess patient demographic characteristics and treatment outcomes. Normally distributed continuous data are expressed as medians and categorical data are expressed as counts and proportions. Survival was calculated using the Kaplan–Meier method and differences in survival outcomes were assessed using a log‐rank analysis. Comparisons among all parameters were analyzed using Student's t‐test. The incidence of new primary lung cancer after the second surgery was calculated as the number of patients who developed new primary lung cancer divided by the total number of patient years.

All data were analyzed using the SPSS software (version 23.0; IBM Corp., Armonk, NY, USA). Significance was set at *p* < 0.05.

## 4 Results

### 4.1 Comparison of Clinical and Pathological Characteristics Between the Groups With and Without New Nodules After the Second Surgery

**FIGURE 2:**
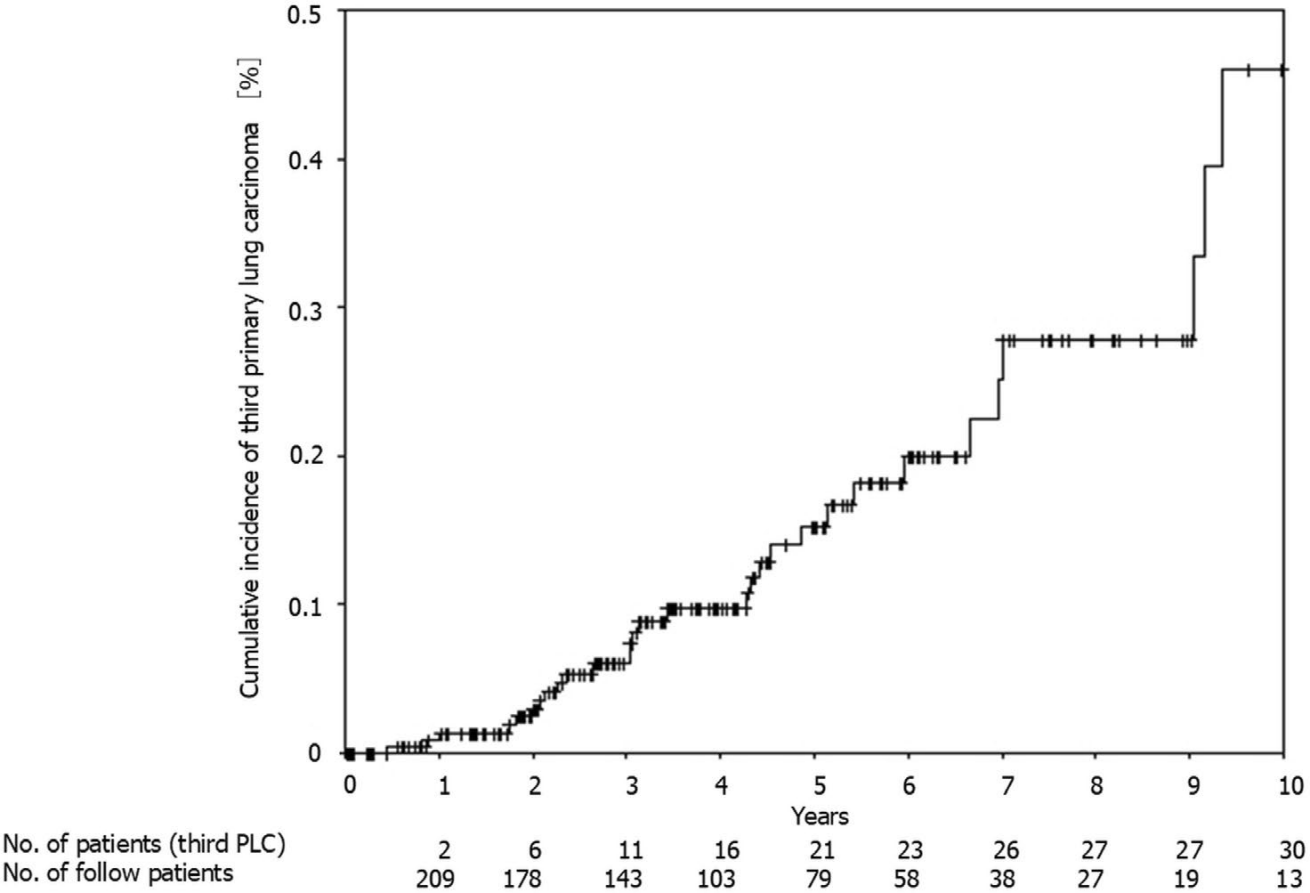
Time to third metachronous primary lung cancer detection. PLC, primary lung cancer.

Table 1 lists the clinical and pathological characteristics of patients in groups A and B. In group B, the median age of the patients was 75 years; 19 (61%) were men, and 25 (81%) presented with preoperative comorbidities. In group B, 21 patients had a history of smoking, with a pack‐year smoking habit of 20.0, and the median interval between the first and second surgeries was 1694 days. In group B, for the second surgery, 10 patients (32%) underwent lobectomy and more than a lobectomy; 25 patients (81%) had pathological stage 1 cancer, and 23 patients (74%) had adenocarcinoma. Significant differences were not observed between the 2 groups regarding age, sex, preoperative comorbidities, smoking history, pack‐years smoked, respiratory function, second surgical procedure, cancer pathological stage during the second surgery, or percentage of adenocarcinoma during the second surgery. Figure 2 shows the period and incidence of new nodules after the second surgery. The incidence rates of new nodules were 0.0091, 0.015, 0.020, 0.024, and 0.029 cases per person 1, 2, 3, 4, and 5 years after the second surgery, respectively.

**TABLE 1:** Patient clinicopathological characteristics before and after the second surgery.

Variables	Group A (*n* = 205)	Group B (*n* = 31)	*p*
Age at second surgery, median [IQR]	72 [66–77]	75 [68–77]	0.76
Men	115 (56%)	19 (61%)	0.58
Preoperative comorbidity	164 (80%)	25 (81%)	0.93
Smoking history	125 (61%)	21 (68%)	0.47
Pack‐year smoking [IQR]	18.0 [0–50.0]	20.0 [0–71.0]	0.12
Duration between the first and second surgery, day [IQR]	1960 [1250–2863]	1694 [1136–2471]	0.19
Respiratory function during the second surgery			
VC, L [IQR]	2.79 [2.32–3.52]	2.64 [2.12–3.13]	0.21
%VC, % [IQR]	95.3 [84.7–109.5]	87.1 [74.1–106.3]	0.48
FEV1, L [IQR]	1.92 [1.59–2.39]	1.82 [1.31–2.20]	0.10
FEVI%, % [IQR]	71.8 [64.3–77.2]	73.9 [64.6–79.6]	0.97
Second surgical procedure			
Segmentectomy or less than a segmentectomy	146 (71%)	21 (68%)	
Lobectomy and more than a lobectomy	59 (29%)	10 (32%)	0.69
Pathological stage during the second surgery			
Stage I	177 (86%)	25 (81%)	
Stage II	14 (7%)	5 (16%)	
Stage IIIA or higher	14 (7%)	1 (3%)	0.40^a^
Pathology during the second surgery			
Adenocarcinoma	166 (81%)	23 (74%)	
Squamous cell carcinoma	28 (14%)	6 (19%)	
Others	11 (%)	2 (7%)	0.38^b^

Abbreviations: FEV1, forced expiratory volume in 1 s; IQR, interquartile range; L, liter; VC, vital capacity.

^a^ Pathological stages I and II versus IIIA or higher.

^b^ Adenocarcinoma versus non‐adenocarcinoma.

### 4.2 Comparison of Clinical and Pathological Characteristics of Patients Who Underwent a Third Surgery and Those Who Did Not

Table 2 presents the clinical and pathological characteristics of patients in subgroups B1 and B2. In subgroup B1, the median age of the patients was 73 years; 13 (57%) were men, and 18 (78%) presented with preoperative comorbidities; 16 had a history of smoking, with a pack‐year smoking habit of 20.0; and the median interval between the first and second surgeries was 1850 days. In subgroup B1, for the second surgery, 5 patients (22%) underwent lobectomy or more than a lobectomy, 19 patients (83%) had pathological stage 1 cancer, and 18 patients (78%) had adenocarcinoma. Significant differences were not observed between the two subgroups regarding age, sex, preoperative comorbidities, smoking history, pack‐year smoking, respiratory function, cancer pathological stage during the second surgery, or percentage of patients with adenocarcinoma during the second surgery. Compared with subgroup B2, subgroup B1 tended to have a lower proportion of patients undergoing lobectomy or more than a lobectomy for the second surgery (*p* = 0.074).

**TABLE 2:** Clinicopathological characteristics of patients who underwent a third primary lung cancer surgery and those who did not.

Variables	Subgroup B1 (*n* = 23)	Subgroup B2 (*n* = 8)	*p*
Age at second surgery, median [IQR]	73 [66–77]	75 [69–77]	0.54
Men	13 (57%)	6 (75%)	0.37
Preoperative comorbidity	18 (78%)	7 (88%)	0.58
Smoking history	16 (70%)	5 (63%)	0.72
Pack‐year smoking [IQR]	20.0 [0–45.0]	119.0 [0–235.0]	0.15
Duration between the first and second surgery, day [IQR]	1850 [1295–2593]	1119 [936–1694]	0.10
Respiratory function during the second surgery			
VC, L [IQR]	2.64 [2.13–3.12]	2.77 [2.49–2.98]	0.59
%VC, % [IQR]	87.1 [74.1–92.8]	88.1 [78.7–97.4]	0.33
FEV1, L [IQR]	1.90 [1.35–2.20]	1.75 [1.39–1.83]	0.47
FEVI%, % [IQR]	76.9 [67.8–79.9]	71.5 [53.1–74.4]	0.15
Surgical procedure			
Segmentectomy or less than a segmentectomy	18 (78%)	3 (38%)	
Lobectomy and more than a lobectomy	5 (22%)	5 (62%)	0.074
Pathological stage during the second surgery			
Stage I	177 (86%)	25 (81%)	
Stage II	14 (7%)	5 (16%)	
Stage ≥ IIIA	14 (7%)	1 (3%)	0.65^a^
Pathology during the second surgery			
Adenocarcinoma	166 (81%)	23 (74%)	
Squamous cell carcinoma	28 (14%)	6 (19%)	
Others	11 (%)	2 (7%)	0.86^b^

Abbreviations: FEV1, forced expiratory volume in 1 s; IQR, interquartile range; L, liter; VC, vital capacity.

^a^ Pathological stage I and II versus IIIA or higher.

^b^ Adenocarcinoma versus non‐adenocarcinoma.

### 4.3 Characteristics of the Third Primary Lung Cancer Surgery

Table 3 describes the details of the third primary lung cancer surgery and postoperative course in subgroup B1. The first and second surgical sites were right‐to‐right in six patients, right‐to‐left in eight, left‐to‐right in three, and left‐to‐left in six. Twenty patients (87%) underwent at least one lobectomy in both the first and second surgeries, and three patients (13%) underwent two lobectomies. The surgical site for the third surgery was the right in 10 patients and the left in 13 patients. The procedures used for the third surgery were wide‐wedge resection in 11 patients (48%), segmentectomy in 8 patients (35%), lobectomy in 2 patients (9%), and CP in 2 patients (9%). The median operative time was 130 min, and the median blood loss was 15 mL. There were 10 postoperative complications, with pulmonary fistula being the most common (six patients), followed by arrhythmia (two patients), respiratory failure (two patients), and myocardial infarction (one patient). One patient had 30‐day mortality; the patient had a myocardial infarction immediately after surgery, was treated in the cardiology department, and died 3 days after surgery.

**TABLE 3:** Intraoperative features for the third surgery group.

Variables	Subgroup B1 (*n* = 23)
First/second surgical site	
Right → Right	6 (26%)
Right → Left	8 (35%)
Left → Right	3 (13%)
Left → Left	6 (26%)
First/second surgery procedure	
WWR + WWR	1 (4%)
Segmentectomy + WWR	1 (4%)
Segmentectomy + Segmentectomy	1 (4%)
Lobectomy + WWR	7 (30%)
Lobectomy +Segmentectomy	10 (43%)
Lobectomy + Lobectomy	2 (9%)
Lobectomy + Completion pneumonectomy	1 (4%)
Third surgical site	
Right	10 (43%)
Left	13 (57%)
Third surgery procedure	
WWR	11 (48%)
Segmentectomy	8 (35%)
Lobectomy	2 (9%)
Completion pneumonectomy	2 (9%)
Lymph node dissection performed in the third surgery	3 (13%)
Length of operation, min [IQR]	130 [96–172]
Blood loss, mL [IQR]	15 [6–40]
Hospital stays in days [IQR]	9 [7–11]
Postoperative complications	10 (44%)
Pulmonary fistula	6 (26%)
Arrhythmia	2 (9%)
Respiratory failure	2 (9%)
Myocardial infarction	1 (4%)
30‐day mortality	1 (4%)
90‐day mortality	1 (4%)

Abbreviations: IQR, interquartile range; WWR, wide‐wedge resection.

### 4.4 Patient Prognoses in the Third Surgery Group (Subgroup B1)

Table 4 lists the pathological findings and prognoses of patients in subgroup B1. The pathological diagnosis was adenocarcinoma in 19 patients, squamous cell carcinoma in 2 cases, and others in 2 patients. The pathological stages were IA, IB, and IIB in 16, 3, and 4 patients, respectively. Seven patients had a pathological stage IB or higher, although none of them underwent postoperative adjuvant chemotherapy. The attending physician's judgment determined that for two of these patients, chemotherapy would be difficult due to a decline in physical strength, and the remaining five patients refused chemotherapy. Recurrence occurred in five patients; four distant recurrences, one local recurrence, and no cases of marginal recurrence. Treatment for recurrence included chemotherapy in three patients, gamma knife surgery in one patient, and surgery in one patient. In three patients, new nodules were observed after the third surgery. Two patients underwent surgery, and one chose the best supportive care as the treatment plan.

**TABLE 4:** Postoperative features of the third surgery group.

Variables	Subgroup B1 (*n* = 23)
Pathology	
Adenocarcinoma	19 (82%)
Squamous cell carcinoma	2 (9%)
Others	2 (9%)
Pathological stage	
Stage IA	16 (70%)
Stage IB	3 (13%)
Stage IIB	4 (17%)
Recurrence case	5 (22%)
Recurrence treatment	
Chemotherapy	3 (13%)
Gamma knife	1 (4%)
Surgery	1 (4%)
Patients with new nodules found during follow‐up	3 (13%)
Treatment of new nodules as described above	
Surgery	2 (9%)
Best supportive care	1 (4%)

**FIGURE 3:**
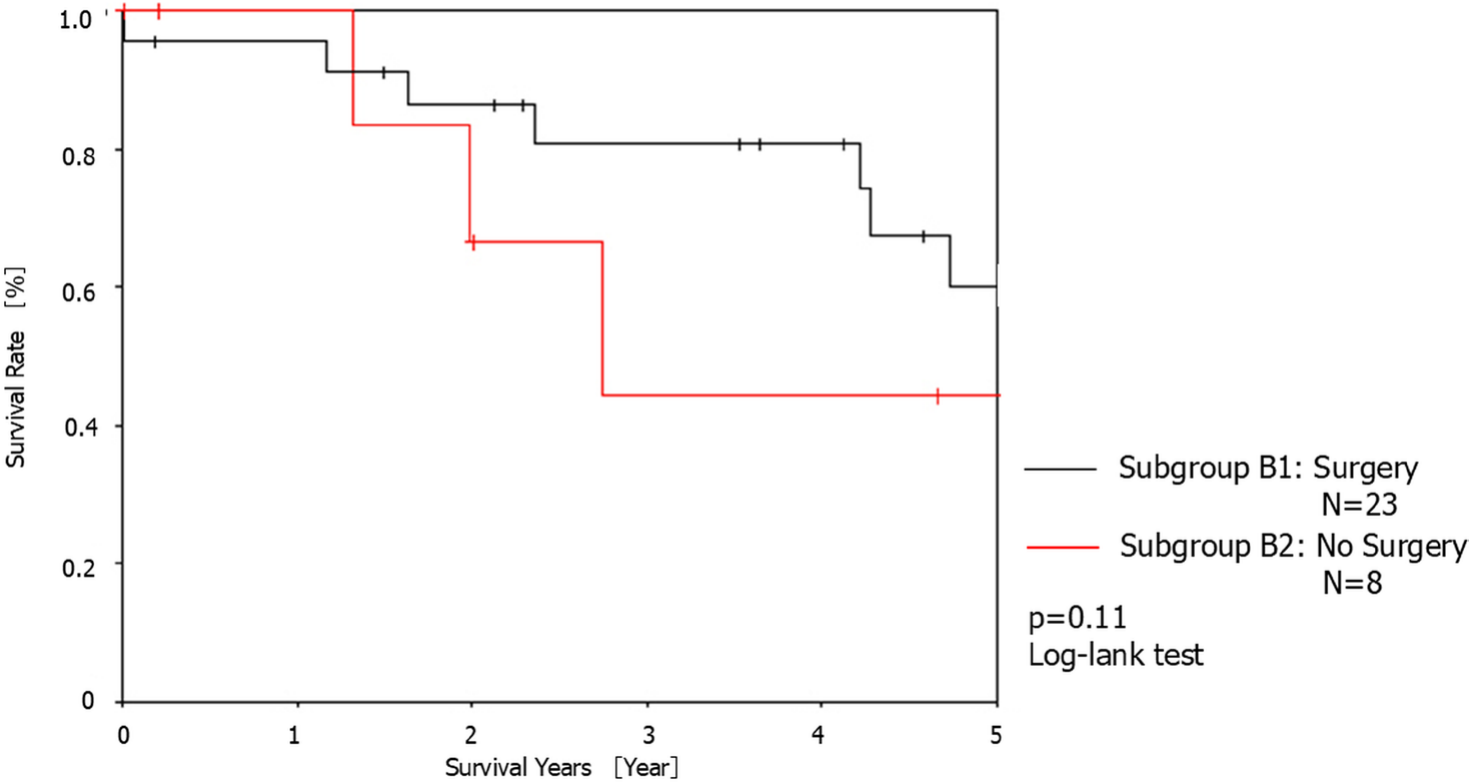
Kaplan–Meier survival curves for the 5‐year overall survival of patients in subgroups B1 and B2.

Figure 3 shows the patient prognoses in group B. The 1‐, 2‐, 3‐, 4‐, and 5‐year survival rates after the second surgery in subgroup B1 were 95.7%, 86.3%, 80.9%, 80.9%, and 60.0%, respectively. The 1‐, 2‐, 3‐, 4‐, and 5‐year survival rates after the second surgery in subgroup B2 were 100.0%, 66.7%, 66.7%, 44.4%, and 44.4%, respectively. No significant differences in prognosis were observed between the two subgroups (*p* = 0.11).

## 5 Discussion

To our knowledge, this was the first study to examine the tolerability of surgery in patients who underwent a third surgery after a second primary lung cancer surgery. We reported a high postoperative complication rate of 44% (10/23) for the third surgery, among which persistent pulmonary fistula was the most frequent at 26% (6/23), death within 30 days was 4.3% (1/23), and the 5‐year survival rate was 60.0%.

Surgical tolerance and complication risks are important issues in primary lung cancer treatment, especially in patients undergoing multiple surgeries. A patient's ability to tolerate surgery depends on a variety of factors, including overall health, lung function, age, and the presence of comorbidities. Preoperative evaluation is particularly important because lung function has a significant impact on postoperative recovery. Previous studies have shown that patients with compromised lung function are at a higher risk of postoperative complications and mortality [11].

The risks associated with a third surgery are related to the nature and complications of the first and second surgeries. For example, if there were complications during the previous surgeries, the risks for those complications may increase during the third surgery. In our study, when a third primary lung cancer was suspected, there was no significant difference between the surgery and non‐surgery groups; however, those in the surgery group tended to have undergone a segmentectomy or less than a segmentectomy during the second surgery. In other words, if a lobectomy was selected for the second surgery, it may not be an option for a third primary lung cancer.

In addition, the tumor location, size, and degree of infiltration into the surrounding tissues should be considered. To determine whether a third surgery is beneficial for the patient, a balance between the risks and benefits must be considered. In this study, a third surgery was performed in 83% (19/23) of the patients with a third primary lung cancer, with a reduced size of less than pulmonary segment resection. Although partial resection was performed in 48% (11/23) of the patients, the median operation time was 130 min, which was considered to be due to the time required for extensive adhesion removal. In addition, the incident rate of persistent postoperative pulmonary fistulas was high (26%), and careful prevention of pulmonary fistulas during surgery is necessary. Although they reported a case of pulmonary metastasis resection, Mills et al. [12] noted that when compared with second surgeries, prolonged air leakage lasted significantly longer after third surgeries.

CP may be required for ipsilateral lung surgery. CP requires advanced surgical techniques, such as extensive adhesion removal and hilar manipulation, making CP have a frequency of intraoperative and postoperative complications of 27.0%–60.0% [13, 14, 15, 16]. In addition, due to reduced blood flow to the bronchial stump caused by lymph node dissection and bronchial artery processing during the initial surgery, the incidence of bronchial stump fistula in CP is high at 7.9%–11.0% [14, 15]. Owing to these complications, the surgical mortality rate of CP is 3.4%–11.0% [13, 14, 15, 16], which is much higher than that of general lung cancer surgery. One of the fatalities in this study involved CP, the third surgery procedure, in which the patient died of myocardial infarction after surgery. However, because the 5‐year survival rate of CP for primary lung cancer is relatively good at 48.9% [14], we believe that the suitability of CP as a third surgery procedure will be a topic for future research.

To our knowledge, we are the first to report that the incidence of new intrapulmonary nodules after a second lung cancer surgery is approximately 2%–3%. The incidence of iatrogenic second primary lung cancer after radical surgery for non‐small cell lung cancer (NSCLC) is estimated to be 1%–2% per year per patient [3]. There were no obvious clinicopathological factors that could have led to the development of a third primary lung cancer after radical surgery for a second primary lung cancer. During outpatient follow‐up, CT should first be performed once a year, and if new nodules are observed, the number of CT follow‐ups should be increased to once every 6 months, or the follow‐up should be changed to intensive follow‐up.

Nonsurgical treatments for third primary lung cancers include SBRT and RFA [17]. The 5‐year survival rate of patients with stage I NSCLC at high risk for lobectomy and who underwent SBRT is 55.6% [18]. The 5‐year recurrence rates after SBRT are as follows: primary tumor, 7.3%; primary tumor involving the lobe (local), 20.0%; regional, 10.9%; local‐regional, 25.5%; and disseminated, 23.6% [19]. RFA has the advantage of being a minimally invasive and precise treatment involving percutaneous intervention under image guidance, and the probability of successful local control is 84.4% for lesions < 20 mm [20]. The 5‐year survival rate of the surgical patients in our study was 60.0%, and we believe that surgery is a tolerable treatment option for third primary lung cancers.

This study has a few limitations. First, this was a retrospective single‐center study. Moreover, no specific protocol was used, and several types of lung cancer cases were included; therefore, the results may not be generalizable. Second, the patient selection may have been limited to those who were originally candidates for surgery. Larger retrospective studies based on protocols from other institutions are required to validate these results.

In conclusion, surgery can be considered an effective treatment modality for third primary lung cancer, notwithstanding associated complications, perioperative mortality, and prognosis.

## Author Contributions

H.I., K.T., M.F., A.H., T.M., and K.S. conceived and planned the experiments. H.I., K.T., Y.W., and A.H. planned and performed simulations. H.I., T.M., and K.S. contributed to the sample preparation. H.I., K.T., and K.S. interpreted the results. H.I. and Y.W. took the lead in writing this manuscript. All authors provided critical feedback and helped shape the research, analysis, and manuscript.

## Disclosure

The authors have nothing to report.

## Ethics Statement

This retrospective study was approved by the Ethics Committee of our institution (E24‐0349) and performed in accordance with the ethical standards in the 1964 Declaration of Helsinki.

## Consent

The Ethics Committee of our institution waived the requirement for individual patient consent due to the retrospective nature of the study.

## Conflicts of Interest

The authors declare no conflicts of interest.

## Data Availability

The data that support the findings of this study are available from the corresponding author upon reasonable request.
